# Serum BAFF and APRIL levels in patients with IgG4-related disease and their clinical significance

**DOI:** 10.1186/ar3810

**Published:** 2012-04-24

**Authors:** Kazuhiro Kiyama, Daisuke Kawabata, Yuji Hosono, Koji Kitagori, Naoichiro Yukawa, Hajime Yoshifuji, Koichiro Omura, Takao Fujii, Tsuneyo Mimori

**Affiliations:** 1 Department of Rheumatology and Clinical Immunology, Kyoto University Graduate School of Medicine, 54 Shogoin Kawahara-cho, Sakyo-ku, Kyoto 606-8507, Japan

## Abstract

**Introduction:**

B cell-activating factor of the tumor necrosis factor family (BAFF) and a proliferation-inducing ligand (APRIL) play a crucial role in B cell development, survival, and antibody production. Here we analyzed the serum levels of BAFF and APRIL and their respective clinical associations in patients with an immunoglobulin (Ig) G4-related disease (IgG4-RD).

**Methods:**

We measured serum levels of BAFF and APRIL in patients with IgG4-RD, primary Sjögren's syndrome (pSS), and healthy individuals. Serum BAFF and APRIL levels in IgG4-RD were assessed for correlations with serological parameters, including Ig, particularly IgG4, and the number of affected organs. Serum BAFF and APRIL levels in IgG4-RD were monitored during glucocorticoid (GC) therapy.

**Results:**

Serum BAFF and APRIL levels in patients with IgG4-RD were significantly higher (*P *< 0.01) than in healthy individuals. The BAFF levels of patients with IgG4-RD were comparable to those of patients with pSS. Although clinical parameters, such as serum IgG4 and the number of affected organs, were not correlated with the levels of BAFF, serum APRIL levels were inversely correlated with serum IgG4 levels (*r *= -0.626, *P *< 0.05). While serum BAFF levels decreased following GC therapy, serum APRIL levels increased during follow-up.

**Conclusion:**

These results indicate that BAFF and APRIL might be useful markers for predicting disease activity in IgG4-RD. Further studies are needed to elucidate the role of BAFF and APRIL in the pathogenesis of IgG4-RD.

## Introduction

Immunoglobulin G4-related disease (IgG4-RD) is a multi-organ disorder characterized by hyper-IgG4 γ-globulinemia, organ infiltration of IgG4-bearing plasma cells, and tissue sclerosis [1-3]. IgG4-RD has recently been recognized as a distinct clinical entity [1-4] comprising a number of disorders, such as type 1 autoimmune pancreatitis (AIP) [3,5-7], sclerosing cholangitis [8], Mikulicz's disease (MD) [1], Küttner's tumor [9], Riedel thyroiditis [10], inflammatory aneurysm [11], tubulointerstitial nephritis [12], and retroperitoneal fibrosis [13,14]. Because the cause of IgG4-RD is unknown, it remains unclear whether this disease should be classified as autoimmune, allergic, or hematologic.

Hypergammaglobulinemia and the existence of disease-related autoantibodies (for example, those against lactoferrin [15], carbonic anhydrase II (CAII) [16], amylase-alpha 2A [17], pancreatic secretory trypsin inhibitor (PSTI) [18], and plasminogen-binding protein peptide [19]) support the hypothesis that autoimmunity may participate in the pathogenesis of IgG4-RD. While the mechanism by which B cells preferentially skew IgG4-class switching is still not determined, recent studies with affected tissue [20,21] have suggested that T helper 2 (Th2) phenotypes of CD4+T cells and regulatory T cells play a crucial role in excessive production of IgG4 and tissue fibrosis.

B cell-activating factor of the tumor necrosis factor (TNF) family (BAFF, also known as B-lymphocyte stimulator (BLyS) or TNF and apoptosis leukocyte-expressed ligand-1 (TALL-1)) and its homolog, a proliferation-inducing ligand (APRIL, also known as TNF-related death ligand 1 (TRDL-1) or TNF and apoptosis leukocyte-expressed ligand-2 (TALL-2), are members of the trimeric TNF family, and both play an essential role in the homeostasis of peripheral B cells [22]. Both cytokines are known to be expressed by a variety of cell types, particularly the myeloid-lineage cells [22,23]. BAFF is synthesized as a membrane-bound or secreted protein, while APRIL exists solely in the secreted form [24]. BAFF binds to three receptors - BAFF receptor (BAFF-R), transmembrane activator and calcium-modulating cyclophilin ligand interactor (TACI), and B cell maturation antigen (BCMA) - which are expressed by B cells, whereas APRIL binds to TACI and BCMA [25].

BAFF and APRIL are thought to mediate the regulation of B cell maturation, survival, CD40L-independent antibody production, and isotype switching through BAFF-R and TACI [22,23,26,27]. Because overexpression of BAFF is known to induce B cell hyperactivation and autoimmunity in mice [28], BAFF has been considered a promoting factor in the pathogenesis of several autoimmune and allergic diseases. In fact, elevated serum levels of BAFF were observed in patients with rheumatoid arthritis (RA) [29], systemic lupus erythematosus (SLE) [30], primary Sjögren's syndrome (pSS) [31,32], inflammatory myositis (IM) [33], systemic sclerosis (SSc) [34], bronchial asthma [35], and atopic dermatitis [36], and serum BAFF levels were associated with their clinical activity. In contrast, overexpression of APRIL has not been associated with autoimmunity in mice but leads to enhanced IgM production, T cell-independent type 2 humoral responses, and T cell proliferation [37]. On the other hand, a lack of APRIL is associated with an increased percentage of CD44^hi^CD62L^low ^effector memory T cells and impaired class switching to IgA [38,39]. Although APRIL has been found to be elevated in patients with autoimmune diseases, including SLE [40], pSS [32], and multiple sclerosis [41], it is still under debate whether APRIL has a role in human autoimmunity, and its circulating levels do not parallel those of BAFF.

The aim of this study was to investigate the contribution of BAFF and APRIL in the pathogenesis of IgG4-RD. We assessed serum levels of BAFF and APRIL by ELISA to analyze their association with clinical manifestations, serological parameters, and treatment.

## Materials and methods

### Patients

All patients were recruited from the Department of Rheumatology and Clinical Immunology, Kyoto University Hospital, Kyoto, Japan. Patients with IgG4-RD (*n = *18; 5 females, 13 males; mean age, 68.6 ± 12.3 years; range, 37 to 79 years) were included in the study. Patients were diagnosed on the basis of clinicopathologic findings [3,4], clinical findings (diffuse/focal enlargement or mass formation, nodular/thickened lesions in one or more organs), elevated serum IgG4 (> 135 mg/dL), and histopathologic features including infiltration of lymphocytes and IgG4-positive (+) plasma cells (IgG4+plasma cells/IgG+plasma cells > 40% and/or IgG4+ plasma cells > 10 cells in 5 high-power fields) with typical tissue fibrosis or sclerosis. None of the patients met the criteria for sarcoidosis, Castleman's disease, Wegener granulomatosis, malignant lymphoma, or pSS. Serum samples were obtained before (*n *= 13) and after (*n *= 5) glucocorticoid (GC) treatment, and the serum from six patients before GC treatment was drawn repeatedly during GC treatment. Ten healthy individuals and thirteen individuals with pSS were enrolled and served as healthy and disease controls, respectively. All of the patients with pSS had signs and symptoms that satisfied the Japanese Ministry of Health criteria for the diagnosis of pSS. All patients and healthy volunteers provided informed consent in accordance with the Declaration of Helsinki, before providing samples. This study was approved by the Medical Ethics Committee of Kyoto University Graduate School of Medicine.

### Measurement of serum levels of BAFF and APRIL

Serum levels of BAFF were determined using an enzyme-linked immunosorbent assay (ELISA) kit (R&D Systems, Minneapolis, MN, USA), and serum levels of APRIL were determined using an ELISA kit (BioVendor Laboratory Medicine, Modrice, Czech Republic). All serum samples were stored at -20°C until use.

### Statistical analysis

Statistical analysis was performed with GraphPad Prism version 5.0a software (GraphPad Software, Inc., San Diego, CA, USA). Nonparametric tests were performed using the Mann-Whitney U test for comparison of the two groups. Correlations were determined by Spearman's correlation. A value of *P *< 0.05 was considered statistically significant. Data are shown as mean ± standard definition (SD).

## Results

### Clinical, laboratory, and histological features of immunoglobulin G4-related disease (IgG4-RD)

We measured serum levels of BAFF and APRIL in 18 patients with IgG4-RD before GC therapy (cases 1 to 13) and after GC therapy (cases 14 to 18) (Table 1). The duration of GC treatment ranged from five days to thirteen years in five patients (cases 14 to 18). Twelve of eighteen (66.7%) patients with IgG4-RD were elderly men. Serum IgG4 levels were > 135 mg/dL in all patients. Biopsy specimens from affected tissues were obtained from 15 of 18 patients, and abundant IgG4-bearing plasma cell infiltration with lymphoplasmacytic infiltrates and sclerosis was observed in all patients. Three patients (cases 7, 14, and 17), who did not agree to undergo biopsy had their conditions diagnosed as IgG4-RD on the basis of hyper-IgG4 γ-globulinemia and typical clinical findings after other diseases were ruled out. Test results of four patients (cases 4, 6, 12, and 18) were positive for rheumatoid factor (RF), those of 10 patients (cases 1 to 5, 9, 12, 14, 16, and 18) were positive for antinuclear antibody (ANA), and those of two patients (cases 3 and 16) were positive for anti-SS-A antibody. Retroperitoneal fibrosis (RPF) was the most frequent clinical manifestation in our cohort.

**Table 1:** Clinical characteristics of patients with immunoglobulin G4-related disease (IgG4-RD).

Case	Age	Serum IgG4 (mg/dL)	RF	ANA	Clinical manifestation	Biopsy/IgG4/IgG ratio
1	73	2890	< 6	40	Lymph, Mikulicz's disease Prostatitis	Prostate/0.60
2	76	2210	< 6	40	Mikulicz's, RPF	Submandibular gland/0.40
3	79	1460	< 6	160 (SS-A)	IN, IP, Küttner's tumor, Lymph, RPF	Submandibular gland/0.73
4	66	1090	30.3	40	AIP, IN, Renal pseudotumor	Kidney/0.70
5	73	592	< 6	320	IN, IP, Lymph, RPF, Sialadenitis	Submandibular gland/0.43
6	62	736	52.0	< 40	Sialadenitis, Lymph	Parotid gland/0.30*
7	77	738	< 6	< 40	Mikulicz's disease	ND.
8	74	389	< 6	< 40	Retro-orbital tumor	Retro-orbital tumor/0.48
9	76	760	< 6	40	AIP, Periureteritis	Ureter/0.38
10	52	383	< 6	< 40	Küttner's tumor	Submandibular gland/0.57
11	70	724	< 6	< 40	Küttner's tumor, Lymph	Submandibular gland/0.40
12	46	675	26.8	80	Mikulicz's disease	Lachrymal gland/0.41
13	37	533	< 6	< 40	Mikulicz's disease	Lachrymal gland/0.50
14	77	655	< 6	80	RPF	ND
15	76	458	< 6	< 40	AIP, RPF	Retroperitoneal/0.70
16	62	315	< 6	40 (SS-A)	AIP, RPF	Pancreas/0.43
17	79	309	ND	< 40	RPF	ND
18	79	1960	65	40	Orbital tumor, Lymph, Lung nodule	Orbital tumor/0.59

AIP, autoimmune pancreatitis; ANA, antinuclear antibody; IN, interstitial nephritis; IP, interstitial pneumonia; Lymph, lymphadenopathy; ND, not determined; RF, rheumatoid factor; RPF, retroperitoneal fibrosis; SS-A, anti-SS-A antibody; *, IgG4-positive plasmacytes > 100/high-power fields.

### Increased serum BAFF and APRIL in IgG4-RD

**Figure 1:**
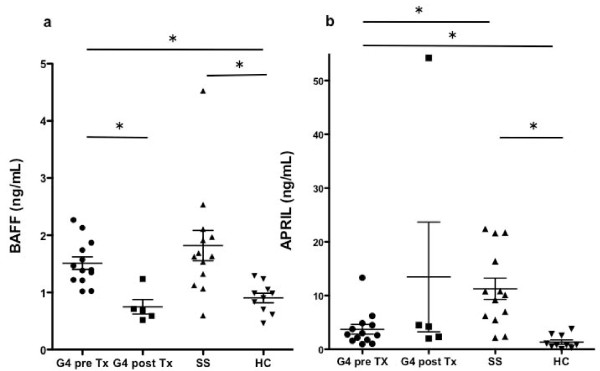
**Serum levels of BAFF and APRIL in IgG4-RD**. **(a) **Serum levels of BAFF in patients with IgG4-RD before (G4 pre Tx) and after (G4 post Tx) treatment with glucocorticoids (GC). **(b) **Serum levels of APRIL in patients with IgG4-RD before (G4 pre Tx) and after (G4 post Tx) treatment with GC. Mean ± Standard deviation (SD) are shown. *, *P *< 0.01. HC, healthy controls; SS, patients with Sjögren syndrome; Tx, treatment.

As shown in Figure 1a, serum levels of BAFF in patients with IgG4-RD before GC therapy (*n *= 13, 1.512 ± 0.393 ng/mL) were significantly higher than those in healthy controls (*n *= 10, 0.904 ± 0.262 ng/mL) (*P *< 0.01), and there were no significant differences in the serum levels of BAFF between patients with IgG4-RD before GC therapy and patients with pSS (*n *= 13, 1.820 ± 0.954 ng/mL) (*P *= 0.383). Serum levels of BAFF in patients with IgG4-RD after GC therapy (*n *= 5, 0.749 ± 0.283 ng/mL) were lower than in those before GC therapy (*P *< 0.01). In contrast, as shown in Figure 1b, serum levels of APRIL in patients with IgG4-RD before GC therapy (*n *= 13, 3.736 ± 3.271 ng/mL) were significantly higher than those in the healthy controls (*n *= 10, 1.327 ± 1.259 ng/mL) (*P *< 0.01), however, unlike BAFF, the levels were significantly lower than those in patients with pSS (*n *= 13, 11.250 ± 7.418 ng/mL) (*P *< 0.01).

### Inverse correlation between serum APRIL and IgG4 in patients with IgG4-RD

**Figure 2:**
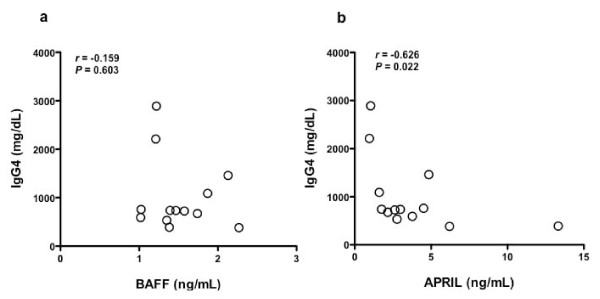
**Correlation between serum BAFF or APRIL and serum IgG4 in patients with IgG4-RD**. Correlation between serum levels of BAFF **(a) **or APRIL **(b) **and IgG4 in patients with IgG4-RD.

While no significant correlation was found between serum levels of BAFF and IgG4 in patients with IgG4-RD before GC therapy (*n *= 13, *r *= -0.159, *P *= 0.603) (Figure 2a), a significant inverse correlation was found for APRIL before GC therapy (*n *= 13, *r *= -0.626, *P *= 0.022) (Figure 2b).

### No correlation between number of affected organs and serum BAFF or APRIL in patients with IgG4-RD

**Figure 3:**
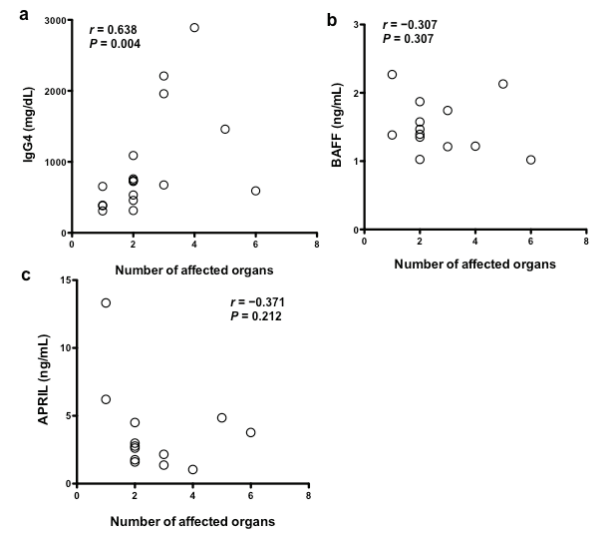
**Correlation between organ involvement and serum IgG4, BAFF, or APRIL in patients with IgG4-RD**. Correlation between the number of affected organs and IgG4 **(a)**, BAFF **(b)**, and APRIL **(c) **in patients with IgG4-RD.

To assess the association between serum BAFF/APRIL and disease severity, we counted the number of affected organs from a list of 10 organs (lacrimal glands, salivary glands, lung, pancreas, kidney, retroperitoneum, lymph node, thyroid glands, prostate, and orbit), which are known to be involved in IgG4-RD. As shown in Figure 3a, a significant correlation between the number of affected organs and serum levels of IgG4 was found in patients with IgG4-RD (*n *= 18, *r *= 0.638, *P *= 0.004). However, as shown in Figure 3b and 3c, no significant correlation was found between the number of affected organs and serum levels of BAFF (*n *= 13, *r *= -0.307, *P *= 0.307) or APRIL (*n *= 13, *r *= -0.371, *P *= 0.212) in patients with IgG4-RD before GC therapy.

### Changes in serum BAFF and APRIL in patients with IgG4-RD during GC therapy

**Figure 4:**
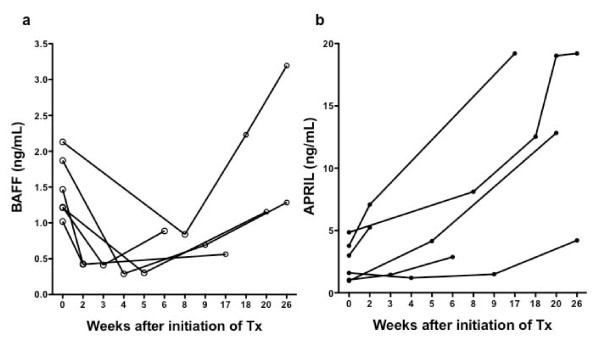
**Changes in serum levels of BAFF and APRIL in patients with IgG4-RD during GC therapy**. Serum levels of BAFF **(a) **and APRIL **(b) **were monitored during and after GC therapy (*n *= 6).

Six patients with IgG4-RD were treated with oral prednisolone (0.6 mg/kg/day) for two weeks, tapering thereafter by 10% of the dose every two weeks, and serum samples were drawn repeatedly to monitor serum BAFF and APRIL during treatment (Figure 4). After treatment, serum levels of BAFF dramatically declined to levels observed in healthy controls in most cases; however, the levels increased again during follow-up (Figure 4a). In contrast, serum levels of APRIL did not decrease during treatment and increased markedly after treatment in most cases (Figure 4b).

## Discussion

This is the first study to demonstrate both an increase in BAFF and APRIL levels in patients with IgG4-RD as well as the differential effects of GC treatment on BAFF and APRIL in patients with IgG4-RD. Promotion of B cell activation, plasmacyte differentiation, and germinal center formation by BAFF and APRIL [42], and ectopic germinal center formation in lacrimal and salivary glands from patients with MD [43] suggest that inappropriate BAFF and APRIL may contribute to progressive plasmacyte infiltration and ectopic germinal center formation in the target organs of patients with IgG4-RD. In addition, it has been demonstrated that BAFF and APRIL enhance class switching to produce IgG4 and IgE in the presence of IL-4 [44,45]. Previous studies have shown that production of Th2 cytokines, such as IL-4, IL-5, and IL-13 was augmented in the tissue of patients with AIP [20]. Therefore, increased expression of both cytokines may contribute to the pathogenesis of IgG4-RD in concert with cognate Th2 cells. In particular, three (cases 3, 4 and 12) out of five patients (cases 3, 4, 10, 11 and 12) with high BAFF levels (> 1.5 ng/mL) had positive test results for autoantibodies (for example, RF or anti-SS-A antibody), which is consistent with previous studies showing that serum BAFF levels were correlated with positive results for serum autoantibodies in patients with RA [29], SLE [30], pSS [31], or IM [33]. BAFF might also play a role in the breakdown of B cell tolerance in patients with IgG4-RD. Recently, it has been reported that serum levels of BAFF were higher in patients with AIP than in those with pancreatic cancer or chronic pancreatitis [46]. The same studies also demonstrated that serum BAFF levels were significantly correlated with serum levels of IgG and IgG4 in patients with AIP, which was not observed in our cohort. The relatively small number of patients in our study or the enrollment of different subsets of patients might explain this discrepancy.

Of interest, we found an inverse correlation between serum APRIL and serum IgG4 levels in patients with IgG4-RD. In SLE, a similar inverse correlation between serum APRIL levels and anti-double-stranded DNA antibody titers has been reported [47], and serum APRIL was inversely associated with disease activity. Thus, APRIL might serve as a protective factor against the progression of IgG4-RD. In our cohort, serum IgG4 levels were significantly correlated with the number of affected organs, clearly indicating that serum IgG4 levels reflect disease severity. Although no significant correlation existed between serum BAFF levels and serum immunoglobulins or the number of affected organs, GC treatment dramatically reduced serum levels of BAFF as well as serum IgG4. In patients with AIP, reduced serum levels of BAFF after 12 weeks of GC treatment have been reported [46], a result similar to our finding. Thus, serum BAFF levels might reflect the clinical activity of this disease. During long-term (up to 26 weeks) follow-up, we observed that the elevated levels of serum BAFF reoccurred in most cases; however, no patients suffered clinical relapse. Further follow-up study will be needed to clarify the relationship between the reoccurrence of elevated serum BAFF and clinical relapse in IgG4-RD. In contrast to the rapid reduction of BAFF by GC treatment, serum levels of APRIL increased over the course of treatment. Similar changes in serum levels of BAFF and APRIL have been reported in patients with GC-treated SLE [47]. Furthermore, no significant correlation was observed between serum BAFF levels and serum APRIL during GC treatment in our patients (data not shown). Thus, universal but distinct mechanisms might exist to control the expression of BAFF and APRIL during GC treatment.

## Conclusions

We demonstrate for the first time that serum BAFF and APRIL levels are increased in patients with IgG4-RD and that the levels of both are indirectly related to clinical activity. Our findings add to the body of knowledge on the role of BAFF and APRIL in the pathogenesis of IgG4-RD. Further longitudinal studies with larger numbers of patients are required to determine the role of the BAFF/APRIL system and to determine whether BAFF and APRIL might serve as therapeutic targets in IgG4-RD.

## Abbreviations

AIP: autoimmune pancreatitis; ANA: anti-nuclear antibody; APRIL: a proliferation-inducing ligand; BAFF: B cell-activating factor of the tumor necrosis factor family; BCMA: B cell maturation antigen; BLys: B-lymphocyte stimulator; CA II: carbonic anhydrase II; ELISA: enzyme-linked immunosorbent assay; GC: glucocorticoid; Ig: immunoglobulin; IgG4-RD: immunoglobulin G4-related disease; IL: interleukin; IM: inflammatory myopathy; MD: Mikulicz's disease; PSTI: pancreatic secretory trypsin inhibitor; pSS: primary Sjögren's syndrome; RA: rheumatoid arthritis; RF: rheumatoid factor; RPF: retroperitoneal fibrosis; SD: standard deviation; SLE: systemic lupus erythematosus; SSc: systemic sclerosis; TACI: transmembrane activator and calcium-modulating cyclophilin ligand interactor; TALL-1: TNF and apoptosis leukocyte-expressed ligand-1; Th 2: T helper 2; TNF: tumor necrosis factor; TRDL-1: TNF-related death ligand 1.

## Competing interests

The authors declare that they have no competing interests.

## Authors' contributions

KK and DK were responsible for the study design, acquisition, analysis and interpretation of data, and manuscript preparation. YH, KK, NY, HY, KO, TF, and TM participated in enrollment of patients and assisted in interpretation of data. All authors have read and approved the manuscript for publication.
